# Evaluating Outcomes of Communication Education for Linguistically Diverse Nursing Home Staff

**DOI:** 10.1093/geroni/igaf122.765

**Published:** 2025-12-31

**Authors:** Maria Roche-Dean, Maria Hein, Yelena Perkhounkova, Carissa Coleman, Frances Yang, Clarissa Shaw, Kristine Williams

**Affiliations:** Kirkhof College of Nursing, Grand rapids, Michigan, United States; University of Iowa, Iowa City, Iowa, United States; University of Iowa, Iowa City, Iowa, United States; University of Kansas Medical Center, Kansas City, Kansas, United States; University of Kansas, University of Kansas Medical Center, Kansas, United States; University of Iowa College of Nursing, Iowa City, Iowa, United States; University of Kansas, Kansas City, Kansas, United States

## Abstract

The ethnic and linguistic diversity of staff working in healthcare has increased, along with the critical role of immigrant workers in meeting workforce demand in long-term service and support settings (LTSS). The focus of this analysis is to compare learning outcomes for nursing home staff who identified a primary language other than English (LOE) to primary English speakers participating in Changing Talk Online Training (CHATO), a pragmatic clinical trial. CHATO is a psychoeducational intervention designed to reduce elderspeak, a form of communication that sounds like baby talk, and has been linked to increased restiveness to care and need for psychoactive medications in LTSS residents with dementia. LOE staff (n = 88) had a greater gain in knowledge than the primary English speakers (n = 1586) with mean scores increasing from 48% on pre-test to 72% on post-test, compared to primary English speakers whose mean scores increased from 57% to 74% (p=.009). Both LOE and primary English-speaking staff expressed positive views regarding CHATO education content. However, LOE participants rated the intervention more difficult than primary English speakers (8.5% v 3.3%, p= .001) which may be associated with technological challenges. Preparing educational content in healthcare that also reaches LOE staff is necessary for a well-prepared workforce; modifications for learning may include digital literacy training and developing organizational policies that support these practices.

